# Bilateral Extra-Cranial Internal Carotid Artery Aneurysms in a Young Adult: A Rare Entity

**DOI:** 10.7759/cureus.16551

**Published:** 2021-07-22

**Authors:** Anshuman Darbari, Rajnish K Arora, Rahul Sharma, Ruhi Sharma, Saravanan Sadhasivam

**Affiliations:** 1 Cardiothoracic and Vascular Surgery, All India Institute of Medical Sciences, Rishikesh, IND; 2 Neurosurgery, All India Institute of Medical Sciences, Rishikesh, IND; 3 Anaesthesiology, All India Institute of Medical Sciences, Rishikesh, IND

**Keywords:** aneurysm, carotid artery, internal carotid artery, extra-cranial internal carotid artery, skull base

## Abstract

Simultaneous bilateral aneurysms of the extra-cranial internal carotid artery (E-ICA) in a patient are one of the rarest lesions. Here, we report the case report of a 19‑year‑old male with bilateral E-ICA aneurysms. His left-sided, expanding aneurysm of E-ICA at skull base was successfully treated with surgical resection and interposition prosthetic graft placement. Successful surgical repair in this challenging field is safe and can be undertaken with an acceptable perioperative complication rate with excellent results.

## Introduction

Aneurysms of the extra-cranial internal carotid artery (E-ICA) are rare lesions. Schechter described only 853 cases in a literature review from 1687 to 1977 [1]. Aneurysms of the internal carotid artery (ICA) at the base of the skull in the infra-temporal fossa are even rarer and <1% of total carotid arterial lesions, and more so, simultaneous bilateral E-ICA aneurysms are rarest of rare [2]. E-ICA aneurysms are usually due to atherosclerosis, post-infectious squeal, traumatic or congenital [3]. Congenital E-ICA aneurysms are commonly associated with connective tissue disorders secondary to a weak muscular wall. E-ICA aneurysm may present in various ways; they may be asymptomatic, may present with headache, dizziness, hearing loss, or other signs due to cranial nerve palsies or pulsatile or non-pulsatile neck swelling. A patient with neurological deficits may present with a various spectrum of cerebrovascular accident (CVA) or Horner's syndrome. These aneurysms may rarely rupture and lead to sudden neurological deficits, otorrhagia, epistaxis or may present with this triad of all features [4].

Besides gold standard conventional angiography, multiple non-invasive diagnostic modalities such as CT or magnetic resonance angiography (MRA) are available to assess the exact size and the extent of the aneurysm with the degree of cerebral circulation completion throughout the Circle of Willis’. The preferred method of repairing these types of aneurysms is to excise the diseased vessel part and do exact end-to-end anastomosis if feasible; otherwise, an interposition graft is required to maintain distal cerebral blood flow. E-ICA can be ligated but carries approximately 30% or higher risk of stroke, depending on the contralateral flow [5]. Despite the increasing role of endovascular therapy and claimed superiority over surgery, open surgical repair is still required, despite being very challenging. Direct surgery is the preferred approach as the degree of rupture, and the endovascular leak chances are very high, especially in large aneurysms with a thrombus producing a compression effect. Various turns and bends in the carotid artery also make it difficult to align in endovascular intervention. Sir Astley Cooper did the first direct intervention of the common carotid artery (CCA) in 1805, but the patient died after 40 hours. The first successful carotid artery aneurysm excision was done in 1952 by Dimtza with end-to-end anastomosis repair [5].

## Case presentation

A 19‑year‑old right‑handed, average-built male was admitted to our hospital because of a pulsatile mass at the left side of his neck. The patient reported that he had noticed this painless, continuously expanding mass one month ago without any provocative trauma. At the same time, he also developed difficulty in swallowing, which was associated with initially solid food but now progressively has increased, even with liquids. The patient also developed hoarseness of voice with left-sided mild ptosis within the last one week. There was no relevant history of any nearby infective lesion, head and neck trauma, or any family history. On clinical examination, a pulsatile, firm, non-tender mass in the left side of the neck was found. On examination, there was also a sensorineural type of hearing loss which was unnoticed by him. No other obvious neurologic symptoms were present and an assessment of the cranial nerves showed no anomalies.

**Figure 1:**
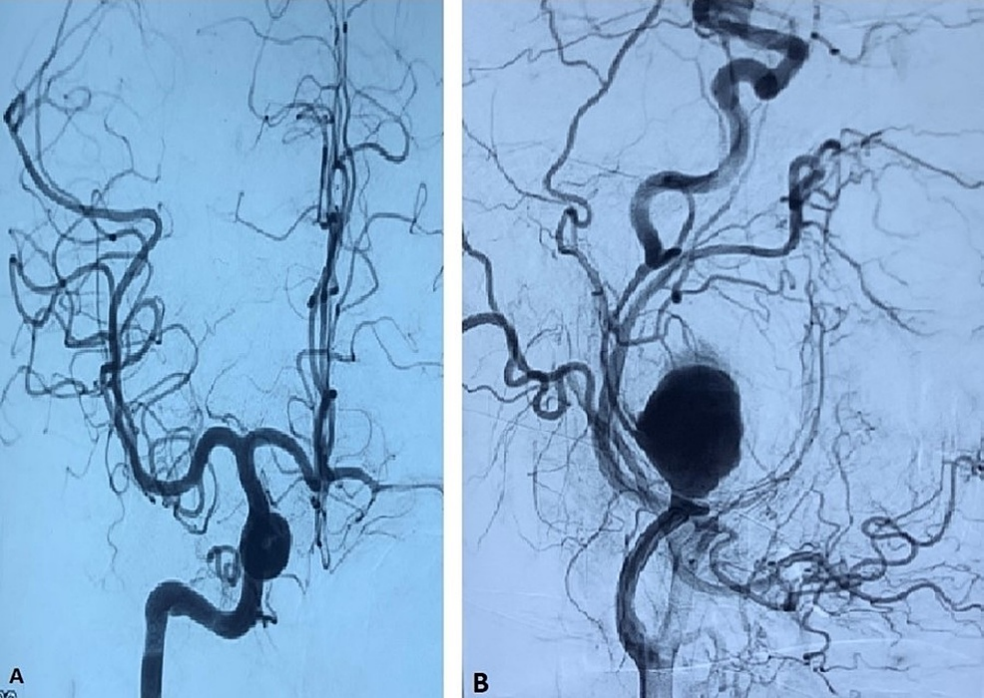
CT angiographic images of patient. A: Right-sided E-ICA aneurysm. B: Left-sided E-ICA aneurysm with displaced and compressed artery. E-ICA, extra-cranial internal carotid artery

After a screening duplex scan revealed a left-sided internal carotid arterial aneurysm, urgent detailed computed tomography angiography (CTA) was done, which showed the presence of aneurysms of the bilateral internal carotid arteries, measuring five centimeters in diameter in the left E-ICA (Figure 1A) and three centimeters in diameter in the right E-ICA (Figure 1B).

**Figure 2:**
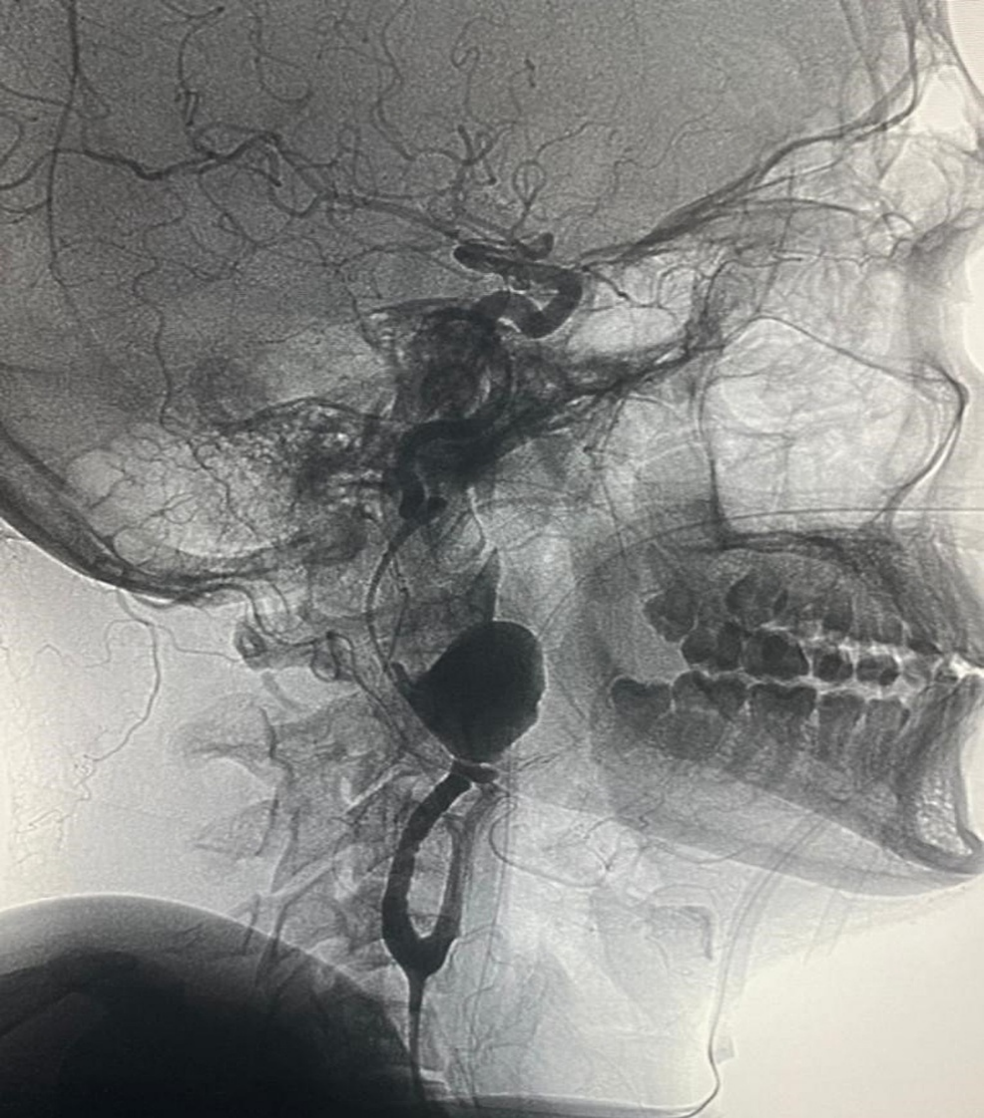
DSA images of left-sided E-ICA aneurysm showing wide-necked aneurysm of ICA with carotid bifurcation. DSA, digital subtraction angiography; E-ICA, extra-cranial internal carotid artery; ICA, internal carotid artery

Conventional angiography was performed with digital subtraction angiography (DSA) and three‑dimensional (3-D) reconstruction to further define the aneurysms and the status of cerebral circulation. The DSA images confirmed bilateral aneurysms of both E-ICAs. The left E-ICA aneurysm measurements were 25 mm × 30 mm, with a cranial‑caudal length of 50 mm (Figure 2).

**Figure 3:**
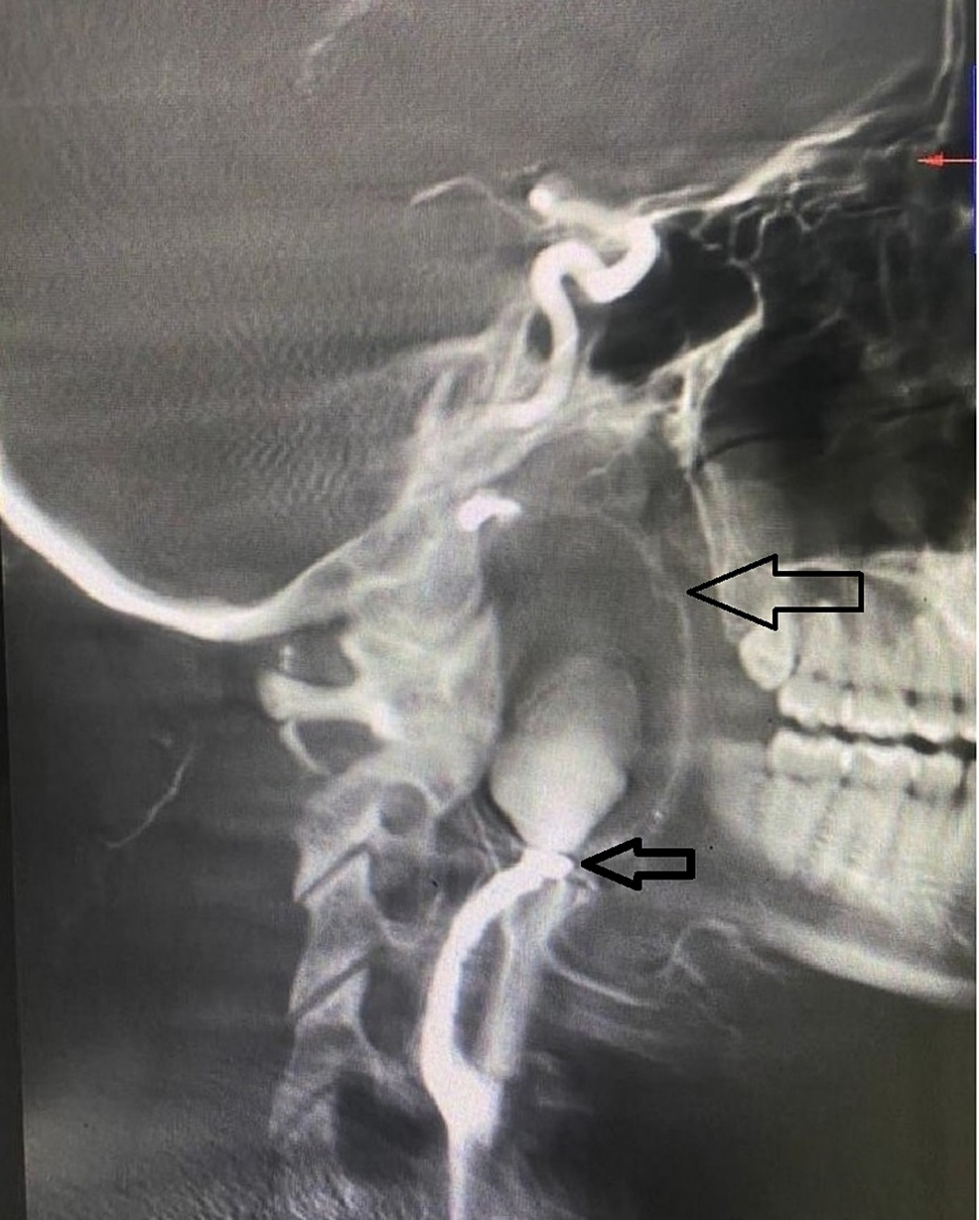
DSA images of left-sided E-ICA aneurysm with two arrows. One upper arrow is pointing to the large outline of thrombus and lower arrow is showing the wide neck of aneurysm. DSA, digital subtraction angiography; E-ICA, extra-cranial internal carotid artery

This aneurysm extended from seven centimeters after the carotid bifurcation in the E-ICA. This probably was a ruptured aneurysm that contained a large thrombus with compression of the left E-ICA near the petrous temporal region with minimal distal flow. As per the Blaisdell line (the line between the mastoid process and angle of the mandible) and segmental classification, the aneurysm was initiated in segment two and extended up to segment three of the E-ICA (Figure 3).

The right E-ICA aneurysm was saccular in shape, measuring 30 mm x 25 mm at segment two. No intracranial aneurysmal disease was identified. In view of simultaneous bilateral aneurysmal disease, the case was thoroughly discussed by a multidisciplinary team consisting of neurosurgeons, vascular surgeons, head and neck surgeons, and intervention neuroradiology. We also suspected a potential connective tissue disorder or vasculitis as a possible cause of this, but relevant blood markers reports were inconclusive. It was decided to do staged procedures for the patient. The left E-ICA aneurysm was large, thrombosed, progressive, angulated and causing pressure symptoms; therefore, urgent surgical intervention was planned. The right E-ICA aneurysm was scheduled for endovascular treatment later in an elective manner, as it was relatively smaller and seems amenable for endovascular intervention.

**Figure 4:**
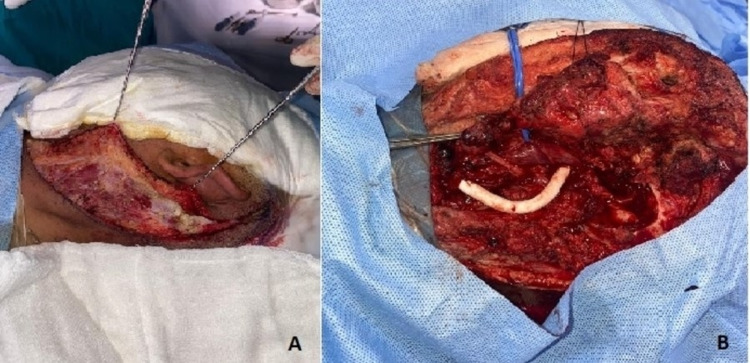
Perioperative pictures. A: Operative incision from neck to post-auricular lateral infratemporal region; B: Completion picture with PTFE prosthetic graft placement in the position. PTFE, polytetrafluoroethylene

The operative procedure was performed under general anesthesia. A standard accepted incision in the neck along the anterior border of the sternocleidomastoid muscle was performed, which extended up to the mastoid (lateral infratemporal approach, Figure 4A). The left CCA was exposed at the carotid bifurcation and hemostatic control was taken at the origin of ICA. The jugular vein, with the Vagus (10th) and Hypoglossal (12th) cranial nerves, were identified, isolated and controlled. The external carotid artery (ECA) with branches was ligated for broader exposure. The ICA was exposed to full length after mastoid exploration with facial nerve translocation in the middle ear. The aneurysm, containing a hematoma, was further isolated up to the petrous part of the skull. However, it became extremely challenging to take distal control of ICA as the aneurysm was extending up to the foramen lacerum. The petrous portion of the temporal bone was opened and temporary using vascular clips, distal control was obtained. After taking control of both sides, the aneurysm was opened. No carotid shunting method was used. Cerebral protection was achieved by avoiding hypotension with control of arterial pressure and systemic heparinization (100 IU/kg). The arterial wall was thickened and friable with the surrounding region of inflammatory changes, and there was a large intraluminal and extraluminal thrombus. After excision of the thrombi present in the aneurysm and diseased vascular region of the vessel, adequate flow from both the proximal and distal aspects of the excised portion of the ICA was observed. Before the operation, we noticed small varicosities in the great saphenous territory of the right lower limb and as the arterial segment loss was very long, a six millimeters carbon-coated polytetrafluoroethylene (PTFE) interposition graft was used within a clamp time duration of 35 min, and both ends anastomosis were performed by six-zero polypropylene sutures using the continuous suturing technique (Figure 4B).

At the completion of operation, the wound was closed in standard fashion. Postoperatively, the patient was given subcutaneous low molecular weight heparin (LMWH) for 72 h in standard doses. Swallowing and feeding were possible on the third postoperative day. Further postoperative recovery was uneventful without any neurologic dysfunction and proper wound healing with a total hospital stay of 21 days.

The patient was discharged on an oral single antiplatelet drug (Tab. acetylsalicylic acid - 150 mg once daily after meals) with a successful outcome. He is in regular follow-up for the last two months after discharge and now is scheduled for the elective endovascular intervention of his right-sided aneurysm.

## Discussion

The E-ICA aneurysm is an infrequent and dangerous lesion. The etiology may be trauma, inflammation, idiopathic, and rarely iatrogenic. Vascular lesions involving the E-ICA at the base of the skull in the petrous segment are extremely rare, as the petrous part of ICA resides in well-protected housing of bone within the carotid canal without vessel branching in this portion. Moreover, it has been also postulated that the petrous part of the ICA is devoid of atherosclerotic changes. However, the anatomic arrangement of the cervico-petrous junction of the ICA may predispose to injury from blunt trauma. As the relatively free cervical portion of the ICA enters the carotid canal, the vessel gets fixed to the skull base, which may be subjected to stretching forces resembling a whiplash-type injury, commonly seen with excessive head movement around the atlantooccipital joint region. This may lead to a dissection of the cervical part of E-ICA and may also have the potential to form a pseudoaneurysm due to a minor leaking effect within the vessel wall. A pseudoaneurysm develops when a leak in the vessel wall dissects within the wall; thereby, creating a swollen, fibrous adventitial tissue capsule. This swelling has the potential to thrombose later. Such injury may result from several mechanisms, including trauma, infections, or inflammatory conditions. Although most often resulting from blunt trauma, E-ICA aneurysms have also been described as rare sequelae of iatrogenic vessel injury during pituitary and sinus surgery as well as from traumatic gunshot wounds. The lack of structural integrity of the arterial wall of a pseudoaneurysm often permits rapid expansion, causing early various neurological problems and eventual fatal rupture. Other reports of rapidly enlarging aneurysms in children and young age groups have been documented with the associated anomalies or idiopathic reasons. Such cases may suggest congenital disorders, including connective tissue diseases such as Marfan’s syndrome and fibromuscular dysplasia. However, the involvement of the E-ICA has not been documented in this age group [6-7].

Differential diagnosis of these aneurysms includes; angular curvature or looping of the ICA, CCA or subclavian artery, carotid body tumour, angioma, cervical lymph nodes enlargement, peritonsillar abscess, inflammation and tumours of the salivary gland, lateral cervical cyst, neoplastic lesions of adjoining structures as palatine tonsil, pharynx, thyroid gland, skin and subcutaneous tissue. Usual symptoms associated with E-ICA aneurysms include headache, nasal congestion, and midface pressure and pain. A ruptured E-ICA aneurysm or dissecting aneurysm may present with otorrhagia, epistaxis, and cranial nerve compression leading to a neurological deficit. Sudden sensorineural hearing loss has also been reported with aneurysms due to compression and dysfunction of the eighth cranial nerve. The presence of an aneurysm in this region may be demonstrated on CT or MR angiographic images after a primary Doppler duplex study. Conventional digital subtraction angiography (DSA) remains the gold standard investigation for diagnosing such vascular lesions [7-8].

The management of petrous E-ICA aneurysms is very challenging. The primary treatment goals for these aneurysms are minimizing the risks of hemodynamic compromise to the cerebrovascular circulation, thromboembolism with stroke prevention, and fatal bleeding. The safety of adequate treatment depends on the etiology, anatomy of the aneurysm, adequate collateral flow to the brain, and the patient's age. Treatment of this kind of aneurysms has been initially limited to rare instances amenable to surgical ligation versus conservative management. Earlier, most case reports favour conservative management or direct ligation of the artery due to complex surgical territory. Open surgical repair and reconstruction of these lesions are advised and documented by Malikov et al. in a series of 13 patients in 18 years. In their entire series, all cases had a unilateral ICA aneurysm [7]. Rosset et al. described another series of 25 cases, over 17 years of ICA reconstructions for EICA aneurysm with surgical reconstruction as a satisfactory therapeutic choice [8]. Aneurysmectomy with the restoration of arterial continuity has been the treatment of choice [9]. The major challenge is proper exposure for vessel control at the skull base. In both the above studies and other isolated case reports, various techniques have been described to get adequate exposure [9-10]. Endovascular treatment has also been proposed as the safer alternative technique, with the sacrifice of the vessel following balloon occlusion testing or endovascular stent placement with distal vessel flow maintenance [11]. The main goal of endovascular management is to exclude an aneurysm with sac thrombus from the arterial circulation with the maintenance of distal cerebral blood flow. Endovascular proximal occlusion methods are considered less invasive than carotid ligation, and the two methods are equally useful [12-13]. There are recent reports of various other new endovascular methods but with very few cases; and therefore, these are not established methods for treatment [14-15]. There are no established guidelines for the treatment of these rare and unusual types of aneurysms [16].

Our patient presented with a rapidly growing pulsatile mass, hearing loss, and early features of Horner syndrome with adjoining area pressure symptoms. There was no history of head and neck trauma in our patient, so we initially suspected vasculitis in light of bilateral presentation. Considering his younger age, we suspected connective tissue disorders, especially fibromuscular dysplasia, but there was no typical image of "string of beads" on angiographies. In our case, treatment options are discussed by a multidisciplinary team. As our patient had a large, expanding aneurysm with acute angulation in E-ICA and possible leakage outside, an urgent open surgically repair was chosen. We faced surgical challenges while exploring the neck region to preserve cranial nerves; also, we had to translocate the facial (seventh) cranial nerve while dissecting the ICA in the middle ear. The arterial exposure in petrous bone was problematic as we elected to cut through the middle ear. The mandibular angle was dislocated and later restored to gain control of the distal arterial end due to this large aneurysm with compression of the vessel's distal flow. However, it was challenging to surgically repair this aneurysm in the “Well" of the petrous bone at the skull base. Still, with proper exposure and multispecialty team help, we were able to repair it successfully.

## Conclusions

An E-ICA aneurysm is an uncommon entity and may be manifested with no or varying symptoms. Safety and long‑term results of appropriate treatment are still evolving due to their rarity and warrant clear indications because of the risk of possible embolization and fatal rupture of these aneurysms. An E-ICA aneurysm at the skull base is a challenging territory for getting distal control and performing the anastomosis during open surgery, which requires various other associated maneuvers. Endovascular therapy may also be considered when expertise is available and in very selected cases only. Surgical reconstructive treatment is feasible with an acceptable risk of stroke and cranial nerve injuries, especially when an aneurysm is located in this challenging territory.
